# Genomic and ecological approaches to identify the *Bifidobacterium breve* prototype of the healthy human gut microbiota

**DOI:** 10.3389/fmicb.2024.1349391

**Published:** 2024-02-15

**Authors:** Chiara Argentini, Gabriele Andrea Lugli, Chiara Tarracchini, Federico Fontana, Leonardo Mancabelli, Alice Viappiani, Rosaria Anzalone, Leonora Angelini, Giulia Alessandri, Giulia Longhi, Massimiliano G. Bianchi, Giuseppe Taurino, Ovidio Bussolati, Christian Milani, Douwe van Sinderen, Francesca Turroni, Marco Ventura

**Affiliations:** ^1^Laboratory of Probiogenomics, Department of Chemistry, Life Sciences, and Environmental Sustainability, University of Parma, Parma, Italy; ^2^Microbiome Research Hub, University of Parma, Parma, Italy; ^3^GenProbio srl, Parma, Italy; ^4^Department of Medicine and Surgery, University of Parma, Parma, Italy; ^5^Laboratory of General Pathology, Department of Medicine and Surgery, University of Parma, Parma, Italy; ^6^APC Microbiome Institute and School of Microbiology, Bioscience Institute, National University of Ireland, Cork, Ireland

**Keywords:** Bifidobacteria, microbiome, genomics, metagenomics, host-microbe interaction

## Abstract

Members of the genus *Bifidobacterium* are among the first microorganisms colonizing the human gut. Among these species, strains of *Bifidobacterium breve* are known to be commonly transmitted from mother to her newborn, while this species has also been linked with activities supporting human wellbeing. In the current study, an *in silico* approach, guided by ecology- and phylogenome-based analyses, was employed to identify a representative strain of *B. breve* to be exploited as a novel health-promoting candidate. The selected strain, i.e., *B. breve* PRL2012, was found to well represent the genetic content and functional genomic features of the *B. breve* taxon. We evaluated the ability of PRL2012 to survive in the gastrointestinal tract and to interact with other human gut commensal microbes. When co-cultivated with various human gut commensals, *B. breve* PRL2012 revealed an enhancement of its metabolic activity coupled with the activation of cellular defense mechanisms to apparently improve its survivability in a simulated ecosystem resembling the human microbiome.

## Introduction

The human body harbors a multitude of microorganisms colonizing a given ecological niche, such as the mouth, pharynx, respiratory system, stomach, intestine, skin, and urogenital tract (Jandhyala et al., 2015). More specifically, with the term human microbiota, we refer to the set of microorganisms that colonize these various human body parts and which, through mutualistic relationships with the host, contribute to the maintenance of its health (Tojo et al., 2014; Bottacini et al., 2017; Hidalgo-Cantabrana et al., 2017; Alessandri et al., 2019). Among the human microbiotas, the intestinal microbiota comprises the most abundant and diverse microbial community of the human body. Bifidobacteria are commonly found as gut commensals throughout an individual's life (Turroni et al., 2011a, 2021), establishing with the host a multitude of trophic and immune interactions (Cebra, 1999; Hooper, 2004). Representatives of *Bifidobacterium bifidum, Bifidobacterium breve*, and *Bifidobacterium longum* are typically the first microbial colonizers of the infant gut microbiota (Turroni et al., 2011b, 2012).

Notably, *B. breve* strains are frequently isolated from stool samples of healthy breastfed infants (Collado et al., 2012) and milk samples of breastfeeding mothers (Khodayar-Pardo et al., 2014), highlighting events of vertical transmission between the mother and her newborn (Milani et al., 2015b). Furthermore, different studies have highlighted how this species is involved in protection against the development of allergies through its impact on the intestinal epithelial barrier, and in modulating the host's immune system (Inoue et al., 2009; Hougee et al., 2010; Bozzi Cionci et al., 2018). In this regard, a previous study showed the role of *B. breve* UCC2003 in the proliferative development of the intestinal epithelial cells during early life (Kiu et al., 2020).

Concerning the establishment of the infant gut microbiota, an *in silico* and *in vitro* analysis revealed the ability of *B. breve* to metabolize simple sugars that may be encountered in the infant gut, such as lactulose, raffinose, maltose, lactose, and glucose (Bottacini et al., 2014). In addition, members of the *B. breve* species can metabolize (certain) Human Milk Oligosaccharides (HMOs), either directly or by cross-feeding, the latter involving other members of the bifidobacterial community such as *B. bifidum* (Egan et al., 2014). These activities clearly show how this species is specialized in colonizing the infant gut (Sela et al., 2008; Turroni et al., 2010; Egan et al., 2014; Lugli et al., 2020a). Interestingly, *B. breve* UCC2003 presents in its genome specific genes involved in the utilization of particular HMOs, such as lacto-N-tetraose (LNT) and lacto-N-neotetraose (LNnT) (James et al., 2016). Moreover, a comparative genomic analysis of the *B. breve* species (Bottacini et al., 2014) highlighted the presence of genes involved in the adaptation to the gastrointestinal environment, such as the production of extracellular structures like fimbria and exopolysaccharides (EPS) (Motherway et al., 2011). Particularly, an EPS produced by *B. breve* UCC2003 was shown to be involved in interactions with the host and protection from a pathogen (Fanning et al., 2012).

Previous research efforts have demonstrated the relevance of “omics” approaches in investigating the genetic makeup and activities of commensal bacteria (Fontana et al., 2022). Notably, analyses of bifidobacterial genomes have paved the way for a burgeoning field known as probiogenomics (Ventura et al., 2009). Specifically, research in the field enhanced our understanding of the diversity, evolutionary processes, and interaction with the human host as well as with other human gut commensals (Ventura et al., 2012; Turroni et al., 2018; Choi et al., 2021). More recently, an *in silico* analysis based on ecological and phylogenomic-driven approach allowed us to identify representative strains of the *Bifidobacterium* genus harboring the human gut microbiota of healthy adults, i.e., *B. longum* subsp. *longum* PRL2022 and *Bifidobacterium adolescentis* PRL2023 (Fontana et al., 2022; Alessandri et al., 2023).

Since *B. breve* has been incorporated as a functional ingredient in various probiotic supplements (Bozzi Cionci et al., 2018), we herein report a genome-based screening aimed at identifying a *B. breve* prototype of the adult gut. The identified representative *B. breve* strain, i.e., PRL2012, was subjected to various omics-based evaluations by means of metatranscriptome analyses when co-cultivated with other bacterial species commonly found in the human microbiome, highlighting particular genetic features of PRL2012 that appear to respond to the presence of and/or sustain interactions with other commensal organisms.

## Results and discussion

### Ecological and phylogenomic-driven identification of the *B. breve* prototype

**Figure 1:**
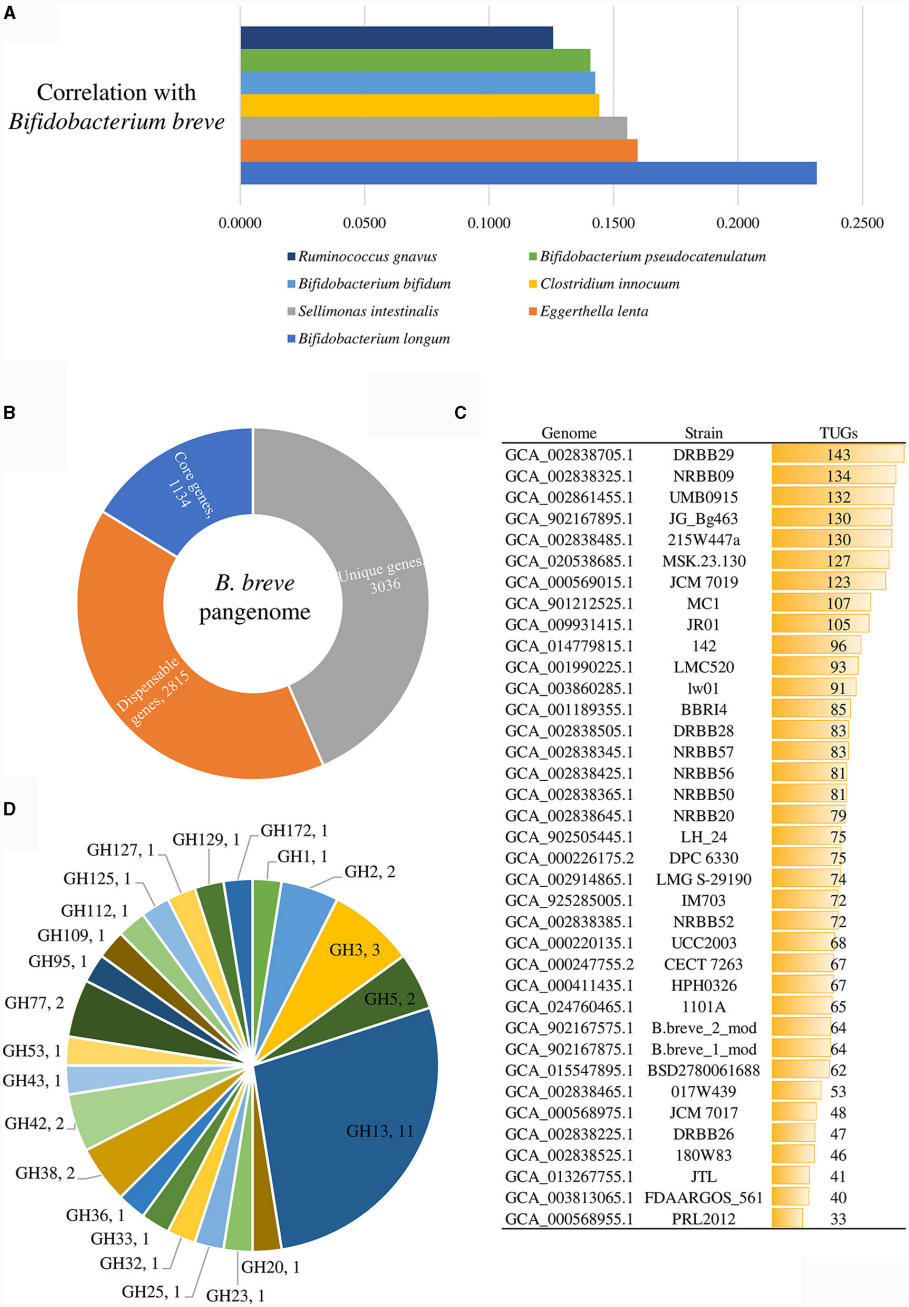
Genomic analyses of representative strains of the 37 identified *B. breve* lineages. **(A)** shows the correlation values of those bacterial species displaying a positive correlation with the *B. breve* taxon, which were statistically significant in shaping the variability of the gut microbiome. **(B)** exhibits the number of core genes (blue), unique genes (gray), and dispensable genes (orange) identified in the pangenome of the *B. breve* species. **(C)** displays the unique gene (TUG) distribution among representative strains of the *B. breve* lineages. **(D)** depicts the glycosyl hydrolase (GH) distribution in PRL2012.

To evaluate the distribution of *B. breve* among the human gut microbiota, an InStrain-based profiling was applied to the genomes of 166 *B. breve* strains retrieved from the RefSeq NCBI database. First, to identify only autochthonous gut bacteria, *B. breve* strains formerly used as probiotics were removed from the analysis. Then, a de-replication procedure was applied using the dRep software among collected genome sequences, allowing the selection of 37 distinct genetic lineages of the *B. breve* species (Table 1 and Figure 1). Finally, the distribution of these identified lineages across microbiomes of healthy individuals was investigated by means of a k-mer based analysis, employing 4,019 gut microbiomes of adults from 82 independent studies (Supplementary Table S1), and an additional 9,505 gut microbiomes of infants (Lugli et al., 2023).

**Table 1:** *Bifidobacterium breve* strain distribution among publicly available datasets of the human gut microbiome.

			**Adult (*****n*** = **4,019)**		**Infant (*****n*** = **9,505)**	
**NCBI code**	**Strain**	**ANI^#^**	**Prevalence**	AxP^*^	**Prevalence**	AxP^*^
GCA_002838325.1	NRBB09	98.36	0.4%	44.05	9.6%	947.21
GCA_901212525.1	MC1	98.10	0.3%	34.17	13.0%	1270.70
GCA_902167875.1	B.breve_1_mod	98.08	0.3%	29.29	9.7%	948.86
GCA_020538685.1	MSK.23.130	98.10	0.3%	26.85	12.6%	1232.20
GCA_902167575.1	B.breve_2_mod	98.30	0.2%	22.01	10.8%	1057.37
GCA_002861455.1	UMB0915	98.10	0.2%	21.97	12.2%	1192.46
GCA_002838525.1	180W83	98.47	0.2%	19.60	13.1%	1285.29
GCA_003860285.1	lw01	98.10	0.2%	19.53	12.4%	1217.15
GCA_902167895.1	JG_Bg463	97.99	0.2%	19.51	11.3%	1105.42
GCA_014779815.1	142	98.14	0.2%	17.09	11.4%	1114.67
GCA_002838705.1	DRBB29	98.14	0.2%	17.09	10.1%	988.02
GCA_925285005.1	IM703	98.47	0.1%	14.70	10.7%	1058.10
GCA_002838365.1	NRBB50	98.46	0.1%	14.70	12.2%	1196.90
GCA_001990225.1	LMC520	98.32	0.1%	14.68	9.9%	972.65
GCA_000568955.1	PRL2012	98.52	0.1%	12.26	10.5%	1036.05
GCA_000247755.2	CECT 7263	98.47	0.1%	12.25	10.7%	1052.78
GCA_015547895.1	BSD2780061688	98.38	0.1%	12.24	10.6%	1038.89
GCA_013267755.1	JTL	98.62	0.1%	9.82	10.6%	1045.76
GCA_002838505.1	DRBB28	98.52	0.1%	9.81	10.2%	1001.59
GCA_000569015.1	JCM 7019	98.13	0.1%	9.77	7.7%	759.46
GCA_002914865.1	LMG S-29190	98.59	0.1%	7.36	10.7%	1051.87
GCA_002838645.1	NRBB20	98.55	0.1%	7.36	8.4%	832.74
GCA_902505445.1	LH_24	98.51	0.1%	7.35	9.9%	972.43
GCA_002838385.1	NRBB52	98.50	0.1%	7.35	8.5%	834.51
GCA_003813065.1	FDAARGOS_561	98.50	0.1%	7.35	9.2%	902.33
GCA_000220135.1	UCC2003	98.48	0.1%	7.35	8.6%	847.26
GCA_000226175.2	DPC 6330	98.46	0.1%	7.35	8.6%	850.29
GCA_002838425.1	NRBB56	98.40	0.1%	7.34	8.5%	836.82
GCA_002838345.1	NRBB57	98.59	0.0%	4.91	8.6%	848.17
GCA_009931415.1	JR01	98.56	0.0%	4.90	9.6%	949.16
GCA_000568975.1	JCM 7017	98.51	0.0%	4.90	8.5%	836.71
GCA_000411435.1	HPH0326	98.44	0.0%	4.90	8.7%	859.76
GCA_001189355.1	BBRI4	98.43	0.0%	4.90	8.5%	837.08
GCA_024760465.1	1101A	98.42	0.0%	4.90	8.6%	847.77
GCA_002838225.1	DRBB26	98.41	0.0%	4.90	8.7%	853.07
GCA_002838485.1	215W447a	98.50	0.0%	2.45	8.2%	807.57
GCA_002838465.1	017W439	98.57	0.0%	0.00	7.2%	713.28

^*^Average × *Prevalence index*. ^#^*ANI between dereplicated genomes*.

These analyses revealed that, when the *B. breve* species was detected, the associated lineage prevalence among infant microbiomes ranged from 7% to 13% for *B. breve* lineages 017W439 and 180W83, respectively (Table 1). Notably, the observed prevalence trend of the *B. breve* genetic lineages resembles a constant cumulative distribution without showing any overrepresented lineages in the infant gut. In contrast, among healthy adult microbiomes, when the *B. breve* species was detected, the lineage prevalence distribution ranged from 0.02% to 0.45% for *B. breve* 215W447a and *B. breve* NRBB09, respectively (Table 1). As expected, this data corroborates with a strong reduction of the overall distribution of members of the *B. breve* species during adulthood compared with infancy (Tarracchini et al., 2023).

Then, an Average x Prevalence index (AxP index) was generated, integrating genetic data produced as ANI values between de-replicated strains and ecological data based on the genetic lineage prevalence among metagenomes (Fontana et al., 2022). The procedure allowed the identification of *B. breve* prototypes in the gut of adults and infants, represented by *B. breve* NRBB09 and 180W83, respectively (Table 1). Therefore, the RefBifSelector tool was employed to identify a representative *B. breve* strain among our local microbial collection (Table 2). The latter multi-omics approach was recently proposed as a tool to select reference strains of a particular (sub)species for *in vitro* analysis (Fontana et al., 2022). Thus, the genome sequence of 11 *B. breve* strains was compared to identify which one possesses the highest percentage of positive scoring matches (PPOS) in relation to the prototype of the adult human gut (Fontana et al., 2022). Accordingly, the genome sequence of the reference strain used as the prototype was *B. breve* NRBB09, whose AxP index was the highest (44.05) when exploring the distribution of the *B. breve* lineages among healthy adults (Table 1). As a result, *B. breve* PRL2012 showed a PPOS of 96.44, representing the optimal reference strain of our local microbial cell collection (Table 2). Interestingly, the selected *B. breve* strain was isolated from the milk sample of a lactating mother, representing a microorganism directly in contact with the newborn during the first days of its life (Mikami et al., 2009; Turroni et al., 2011a). This identified RefBif of the *B. breve* species was then further investigated through *in silico* genomic screenings and *in vitro* experiments to assess its interactions with other elements of the intestinal microbiota and with its host.

**Table 2:** Identification of a genetically representative strain to the *B. breve* NRBB09 prototype.

**Query (NCBI code)**	***B. breve* strain**	**ANI**	**Average PPOS (percentage of positive scoring matches)**	**ANI × Average PPOS**
GCA_000568955.1	PRL2012	98.2	96.43	9469.6
GCA_000568895.1	2L	98.2	96.43	9468.8
GCA_000568875.1	31L	98.0	96.18	9422.1
GCA_002075865.1	1900B	98.2	95.62	9389.6
local	676B	98.0	95.65	9375.8
GCA_000569055.1	689b	98.0	95.64	9372.5
local	158B	98.1	95.51	9368.8
GCA_002076075.1	1889B	98.1	95.51	9367.9
GCA_002076055.1	1891B	97.7	95.31	9308.7
GCA_016648985.1	M1D	97.6	95.28	9300.5
GCA_016648955.1	PRL2020	97.7	95.23	9299.8

### Understanding the molecular cross-talk between gut commensals and *B. breve*

As previously reported, the ability of *B. breve* PRL2012 to interact with other gut commensals, such as *B. bifidum* PRL2010, *B. adolescentis* 22L, and *B. longum* subsp. *infantis* ATCC15697, was investigated under *in vivo* conditions, analyzing its transcriptome from murine cecum samples following its supplementation to conventional BALB/c mice (Turroni et al., 2016). Accordingly, the effects of bifidobacterial co-association between strains were explored by the administration of multiple strains, while their transcript expression was profiled by using custom-made arrays representing 98% of the genes harbored (Turroni et al., 2016). Among the four bifidobacterial species tested, PRL2012 showed the highest cross-talk index, i.e., representing the total number of genes whose expression was modulated by the presence of PRL2012, suggesting that its transcriptome was highly affected by interactions with other bifidobacterial strains (Turroni et al., 2016). Interestingly, the re-analysis of the overexpressed genes unveiled that when PRL2012 was administered to mice at a daily dose of 10^9^ colony-forming units, following the cluster orthologs gene (COG) classification, a large part of the up-regulated genes (≥2-fold change) was predicted to be associated with amino acid and carbohydrate transport and metabolism (>30% of the up-regulated genes). Similarly, administration of a combination of multiple bifidobacterial species to mice revealed a tendency of the PRL2012 strain in the up-regulation of COGs related to translation and replication, followed by amino acid and carbohydrate metabolism-related genes. Notably, cross-feeding interactions between members of the bifidobacterial species and the *B. breve* taxon, as well as with other gut commensals such as *Faecalibacterium prausnitzii* and *Eubacterium hallii* have previously been explored (Egan et al., 2014; Moens et al., 2016; Bunesova et al., 2018). Nonetheless, a complete understanding of the cross-talk among *B. breve* and other microbial taxa that coexist in the same intestinal environment is still lacking.

A correlation analysis between the commensals of the above reported 4,019 gut microbiomes of adults was performed to assess if *B. breve* PRL2012, as a proposed *B. breve* prototype of the adult human gut, is able to persist in the adult human gut and to exert benefits to the host not only during weaning but also in adulthood. This correlation analysis pinpointed those species that frequently coexist within the same ecosystem with the *B. breve* taxon. In total, 66 microbial species exhibited a statistically significant positive correlation with *B. breve* (Benjamini–Hochberg, FDR *p* < 0.05) (Supplementary Table S2). Subsequently, a Principal Coordinate Analysis (PCoA) was performed to delve more deeply into the identification of those bacterial species that are significant in terms of their impact on the human gut microbiota variability. After normalizing the data, it became evident that out of the 66 bacterial species displaying a positive correlation with the *B. breve* taxon, only 20 of them were statistically significant in shaping the variability of the gut microbiome (R^2^ > 0.2, FDR *p* < 0.01) (Supplementary Table S2). Among them *Bifidobacterium longum, Eggerthella lenta, Sellimonas intestinalis, Clostridium innocuum, Bifidobacterium bifidum, Bifidobacterium pseudocatenulatum*, and *Ruminococcus gnavus* fit into the eight species with the highest correlation values with *B. breve*, illustrating a distinct interconnection between the bacteria influencing the diversity of the human gut and *B. breve* (Figure 1A).

### Genetic features of *B. breve* PRL2012

To obtain a comprehensive view of the genetic traits of PRL2012, its genome sequence was decoded employing a combination of short- and long-read technologies (see Materials and Methods), resulting in a complete genome sequence (i.e., a single contig representing a circular chromosome). Then, a comparative genomic analysis of the *B. breve* species was performed, including the predicted encoded proteome of each representative strain of the above-identified 37 lineages (Table 1 and Figure 1). In this manner, pangenome and core-genome analyses of this taxon were undertaken following a previously described method based on Clusters of Orthologous Groups (COGs) (Lugli et al., 2018, 2019, 2020b). The analysis resulted in the identification of 6,985 COGs, representing the pangenome of the representative strains covering all 37 lineages. Among them, 1,134 COGs were shared between the 37 *B. breve* genomes, thus representing their core genome (Figure 1B). Furthermore, truly unique genes (TUGs) of each strain were detected with an average of 82 TUGs per genome. Interestingly, *B. breve* PRL2012 was shown to be the strain with a lowest number of TUGs (*n* = 33) when compared to members of the other lineages (Figure 1C). Accordingly, the number of genes harbored by PRL2012 was shown to be lower than that of all other lineage-representative *B. breve* strains, suggesting a stable genome structure typical of microorganisms specialized in living in a defined ecological niche. In this context, two putative genes encoding carbohydrate-active enzymes were found among the identified PRL2012-specific TUGs.

An *in silico* prediction of the PRL2012 genome, based on the Carbohydrate-Active enZYmes (CAZy) database (Drula et al., 2022), revealed that 40 genes were identified as glycosyl hydrolases (GHs) encompassing 23 different families (Figure 1D). Among them, most of the identified GHs (*n* = 11) were predicted to encode members of the GH13 family, encompassing predicted α-glucosidase, amylase and pullulanase enzymes, which would be consistent with previously described starch- and pullulan-degrading activities of *B. breve* (Ryan et al., 2006). Notably such genes were also identified in the core genome of the species through comparative genomics analyses (Bottacini et al., 2014). On the other hand, additional GH family members encoded by the genome of PRL2012 were identified to process a wide variety of carbohydrates, such as α-galactosidase (GH36), β-galactosidase (GH2 and GH42), lacto-N-biosidase (GH20), α-mannosidase (GH38 and GH125), and β-L-arabinofuranosidase (GH127) (Figure 1). The deduced glycobiome of PRL2012 displays an overall glycan degradation capability, which is in line with that of other *B. breve* strains (Bottacini et al., 2014). To better understand *B. breve* PRL2012's actual ability in processing glycans, we set up *in vitro* growth experiments with various carbon sources.

### Ability of *B. breve* PRL2012 to metabolize carbohydrates

**Figure 2:**
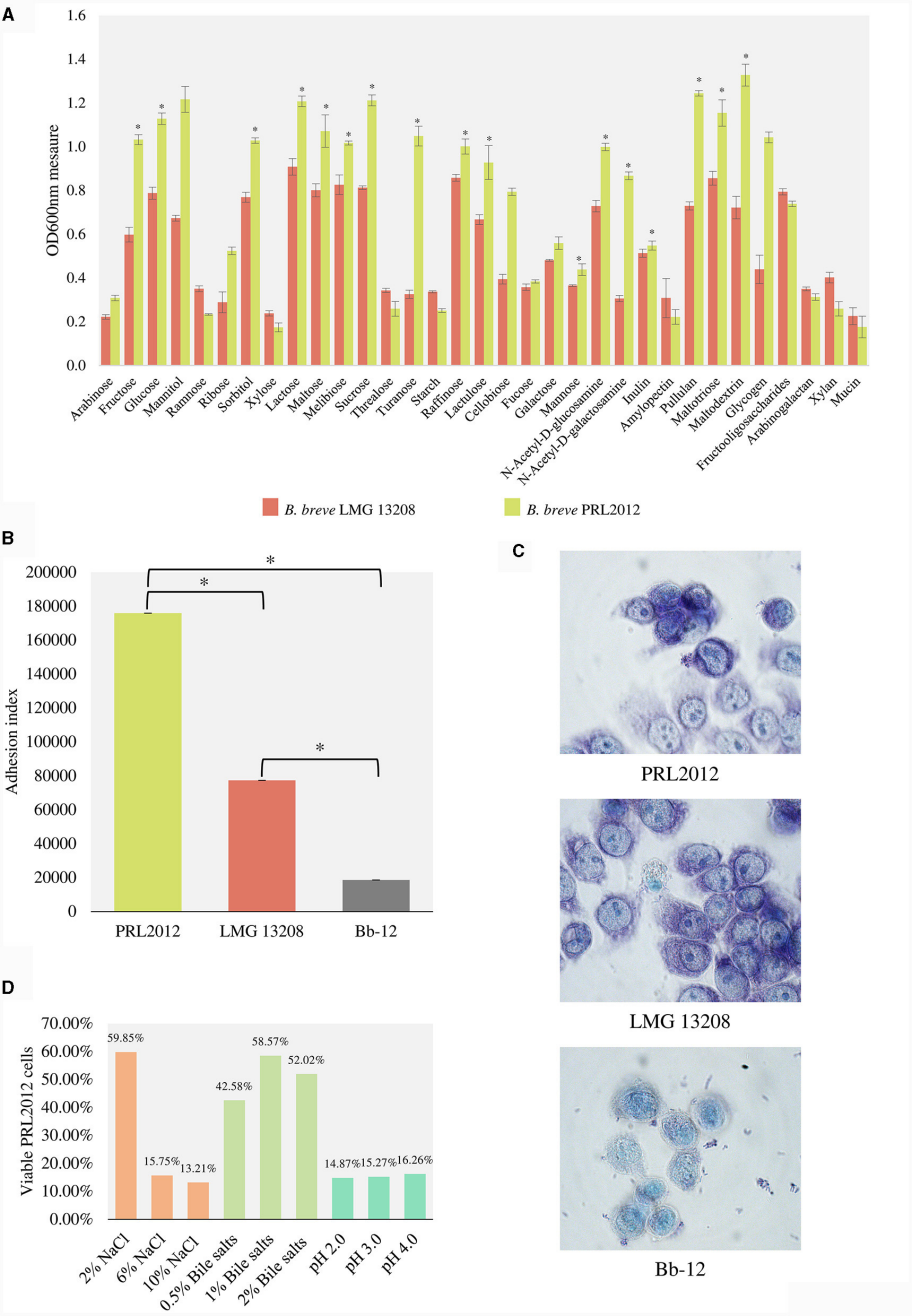
*In vitro* evaluation of the capability of the PRL2012 prototype. **(A)** shows the growth performance of *B. breve* PRL2012 and LMG 13208 on different carbohydrates as measured by optical density at 600 nm (OD_600nm_). **(B)** displays the adhesion index of *B. breve* PRL2012 and LMG 13208 and *B. animalis* subsp. *lactis* Bb-12 cells to HT29-MTX cells monolayers. The vertical bars indicate standard deviations, and the asterisks indicate Mann–Whitney test *p* < 0.05. Data are expressed as the average of the obtained triplicates. **(C)** exhibits light microscopic images of HT29-MTX monolayer cells as observed with Giemsa staining of *B. breve* PRL2012 and LMG 13208 and *B. animalis* subsp. *lactis* Bb-12 cells. **(D)** Tolerance of *B. breve* PRL2012 strain toward human gut challenges. The Y axis represents the percentage of the viable PRL2012 cells.

Growth capabilities of *B. breve* PRL2012 on different carbohydrates were evaluated and compared with those obtained for the *B. breve* type strain, i.e., LMG 13208 (Figure 2A). The choice of the latter strain was due to the results of *in silico* tracking analyses, which emphasized that the type strain of the species was less prevalent in healthy individuals compared to PRL2012 (Table 1). These carbohydrates (see Materials and Methods) include both plant- and host-derived glycans that are commonly found in the adult human gut microbiota (Chassard and Lacroix, 2013). To evaluate the carbohydrate growth capabilities of *B. breve* strains, we used a carbohydrate-free based MRS medium, which was supplemented with one of 33 different sugars, as the unique carbon source (Supplementary Table S3 and Figure 2A). Results of each growth-profiling experiments were processed using a Mann–Whitney test with Benjamini–Hochberg correction (cut-off *p* < 0.05), highlighting statistically significant differences in growth performances between the two *B. breve* strains. In detail, PRL2012 shows broader metabolic capabilities on different monosaccharides and disaccharides, such as fructose, glucose, sorbitol, lactose, maltose, melibiose, sucrose, turanose, and raffinose (final OD value > 1.0, all Benjamini–Hochberg corrected *p* < 0.05) (Supplementary Table S3). In addition, PRL2012 shows appreciable growth on different complex monosaccharides, disaccharides and polysaccharides, such as N-Acetyl-D-galactosamine, N-Acetyl-D-glucosamine, lactulose, pullulan and maltodextrin (final OD value from 0.4 to 1.3; all Benjamini–Hochberg corrected *p* < 0.05) (Supplementary Table S3 and Figure 2A). These data demonstrate significantly broader growth performances of *B. breve* PRL2012 compared with the type strain of this species, corroborating our *in silico* analyses on PRL2012.

### Evaluation of the adhesion features of PRL2012 toward eukaryotic cells

To support PRL2012 strain resilience in the human gut, its adhesive performances to human intestinal mucosal cells were evaluated using a previously described methodology (Guglielmetti et al., 2008; Turroni et al., 2013; Rizzo et al., 2023), seeding cells of *B. breve* PRL2012 on human colon intestinal HT29-MTX monolayers. After multiple washes with PBS, the number of microbial cells attached to the HT29-MTX monolayer was assessed in order to calculate the adhesion index, which represents the number of bacterial cells adhering to 100 HT29-MTX cells (Guglielmetti et al., 2008). Interestingly, a higher adhesion index was observed for *B. breve* PRL2012 (adhesion index of 176,000 ± 8) compared to *B. breve* LMG 13208 (adhesion index of 77,333 ± 6) (Mann–Whitney test *p* < 0.05) (Figures 2B, C). Furthermore, adhesion of the *Bifidobacterium animalis* subsp. *lactis* Bb-12 strain, commonly used as a health-promoting microorganism in many probiotic supplements (Garrigues et al., 2010; Jungersen et al., 2014), was assayed. Results indicated an adhesion index of Bb-12 < 20,000 (Figures 2B, C), highlighting the markedly high adhesion yield of *B. breve* PRL2012 cells toward human cell monolayers. In addition, an adhesion assay on mucin was performed involving the two *B. breve* strains, highlighting an enhanced relative adhesion to mucin of *B. breve* PRL2012 when compared to the type strain LMG 13208, i.e., 73.4% and 66.1%, respectively (Figure 2). Additionally, PRL2012 demonstrates resilience toward typical adverse conditions that bacteria must confront during the passage through the gastrointestinal tract (Figure 2D) (see Supplementary material). These data confirm our previous observations, suggesting that *B. breve* PRL2012 is ecologically adapted to the human gut microbiota ecosystem and probably possesses genes involved in contributing to its (long term) colonization ability of the human intestinal mucosa.

### Investigating the molecular interplay between PRL2012 and typical human gut microbiota member

To assess the interactions between PRL2012 and bacterial species that are typically associated with the adult human gut microbiota, we performed co-cultivation experiments of PRL2012 with those commensal bacteria previously identified to be significant in shaping the human adult gut microbiota and, at the same time, were significantly correlated with *B. breve* in the same environment (Supplementary Table S2). In detail, a PCoA analysis was performed using the abundance data of the microbial species inhabiting the gut microbiomes of 4,019 healthy adults, allowing the identification of those bacterial species that are considered to be affect the human gut microbiota biodiversity. Then, identified bacteria correlating with the presence of *B. brev*e were selected (Supplementary Table S2). Thus, six co-cultivation assays were performed using *B. breve* PRL2012 co-incubated with *B. bifidum* PRL2010, *B. longum* PRL2022, *B. pseudocatenulatum* LMG 10505, *Clostridium innocuum* 107F, *Eggerthella lenta* 180F, and *Ruminococcus gnavus* DSM 114966. Since exploring the molecular interplay between two bacterial strains is rather limited to understand the complex relationships occurring within the gut microbiota, we performed a co-cultivation experiment involving all the above-mentioned microbes, along with PRL2012. A quantitative PCR (qPCR) approach was applied to evaluate the bacterial load of each species from co-cultivation experiments. This qPCR analysis highlighted that PRL2012 can grow in all co-culture experiments with various tested gut commensals (Supplementary Figure S1), since PRL2012 cells identified always yielded a genome copy number higher than 2 × 10^8^ per mL at the end of the co-cultivation experiments. Shotgun metatranscriptomics was then performed in each co-cultivation experiment to explore the interactions of PRL2012 with other bacterial commensals. Compared to the reference condition (PRL2012 grown in a monoculture), PRL2012 revealed a notable impact on its transcriptome (number of genes whose expression was significantly modified) only when it was grown in combination with other bacteria (120 up-regulated genes vs. 42 down-regulated genes) (Supplementary Table S4).

**Figure 3:**
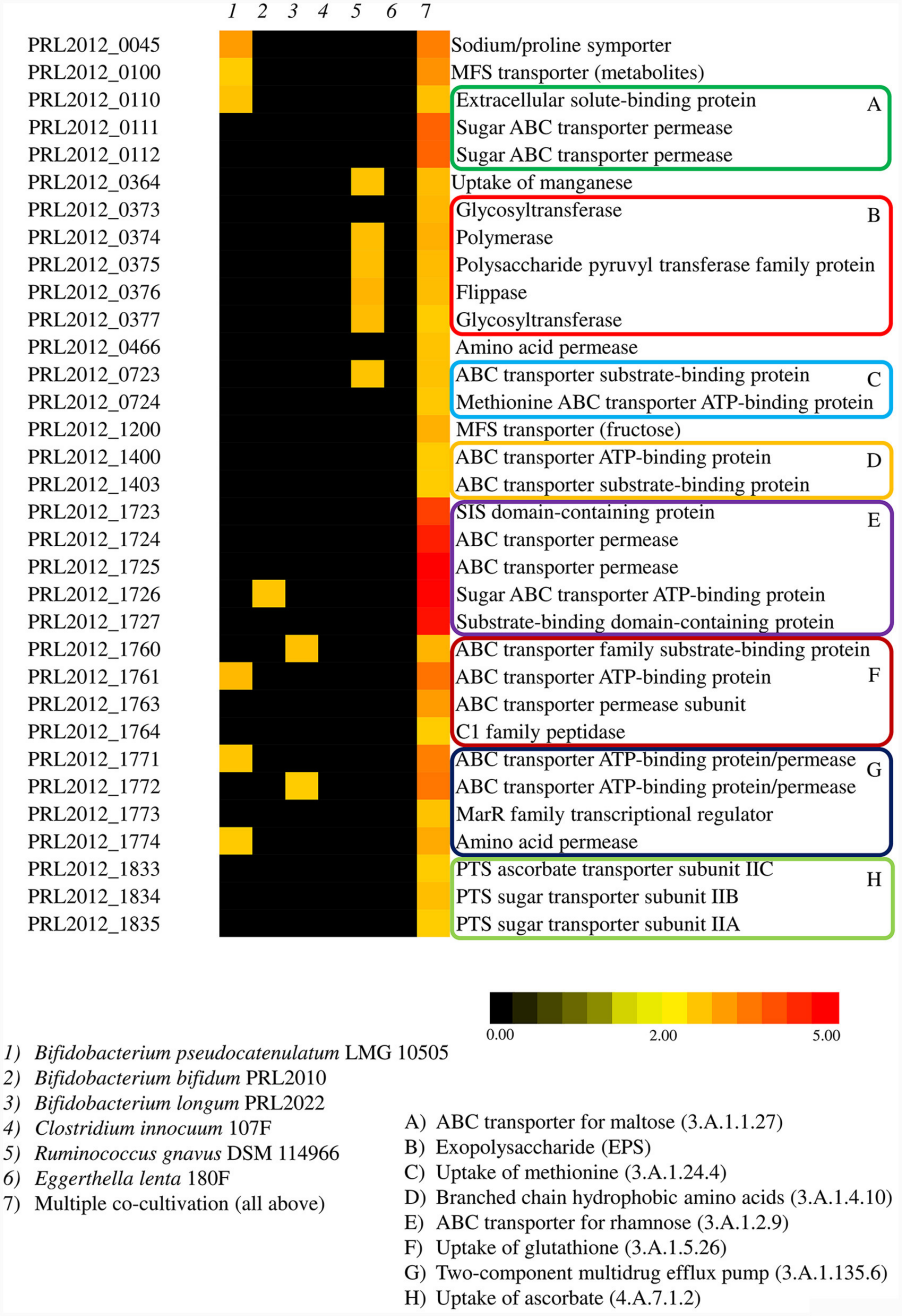
Transcriptome analyses of PRL2012 when co-cultivated with other human gut commensals. The heatmap displays significant overexpressed genes of PRL2012, reporting their function (on the right), co-cultivated commensals (on the top), and the fold change in the color legend (on the bottom).

Transcriptomic data of PRL2012 co-cultivated with six different human gut commensals revealed an enhancement of the transcription of multiple genes related to bacterial metabolism, specifically those predicted to be involved in carbohydrate metabolism (Supplementary Table S4), which is a common strategy of bacteria competing for the same resources (Khoroshkin et al., 2016). The identified up-regulated genes belong to various transporter systems encoded by genes that are scattered across the PRL2012 chromosome, including, among the most significant, a predicetd ABC transporter for rhamnose (3.A.1.2.9), as well as a putative ABC transporter for maltose (3.A.1.1.27) (Figure 3, Supplementary Table S4). Furthermore, genes encoding putative multicomponent transporters composed and predicted to be involved in the uptake of methionine (3.A.1.24.4), glutathione (3.A.1.5.26), ascorbate (4.A.7.1.2) and branched chain hydrophobic amino acids (3.A.1.4.10) were shown to be up-regulated (Figure 3, Supplementary Table S4). Additionally, transporters composed of a single subunit, which were predicted to be dedicated to the uptake of proline (2.A.21.2.5), manganese (2.A.55.3.3), fructose (2.A.1.7.17), and various metabolites (2.A.1.6.10) and amino acids (2.A.3.3.22), were shown to be up-regulated following the co-cultivation of PRL2012 with other gut commensals (Figure 3, Supplementary Table S4). Overall, the ability to shift its carbohydrate and amino acid metabolism among various substrates may be reflective of an adaptive strategy enabling PRL2012 to successfully establish itself in the gut microbiota of adults.

Interestingly, genes directly linked to metabolic processes were not the only genes that underwent an upregulation upon co-cultivation of PRL2012 with multiple bacteria. A two-component multidrug efflux pump (3.A.1.135.6) of PRL2012 was also found to be up-regulated, probably as a defense system when sharing the same environment with multiple bacteria (Supplementary Table S4). Also, a cluster of six genes putatively encoding a biosynthetic machinery to produce an exopolysaccharide (EPS) were activated in co-culture, revealing a possible enhancement in production of these extracellular macromolecules that have previously been shown to play a key role in microbe-microbe interactions (Neville et al., 2013).

## Conclusions

An extensive body of scientific literature supports the well-established benefits of bifidobacteria toward the human gut, which include promoting the integrity of the intestinal barrier, enhancing gut homeostasis, and aiding the development of the host's immune system (Alessandri et al., 2019). In this regard, *B. breve* stands out as one of the 17 most prevalent bacterial species residing in the gastrointestinal tract of infants (Lugli et al., 2023). Thus, we applied an innovative ecological and phylogenomic-driven approach to identify the most representative phylotype of this species within the human gut of infants and adults (Fontana et al., 2022; Alessandri et al., 2023). From our local microbial collection, *B. breve* PRL2012 was shown to exhibit the closest genetic resemblance to the phylotype of the species, while comparative genome analysis revealed a stable genome structure with a very small number of unique genes, two of which were predicted to be involved in carbohydrate metabolism. In this context, the metabolic capabilities of PRL2012 to swiftly adapt its carbohydrate metabolism to compete with other members of the human microbiota, as well as its ability to activate cellular defense mechanisms, were validated through a metatranscriptomic investigation in combination with other intestinal commensals. Similarly, its crosstalk ability was supported by the results of *in vitro* experiments demonstrating that PRL2012 has the capacity to survive and potentially colonize the intricate ecological niche of the human intestine, interacting with the epithelium, adhering to the host mucosa, and withstanding stressful conditions commonly encountered in the intestinal environment.

Based on the results achieved from our *in silico* and *in vitro* analyses, PRL2012 represents a promising candidate to be used as health-promoting bacterium. Data suggest that its ecological adaptation to the human gut microbiota ecosystem contributes to its ability for long-term colonization of the human intestinal mucosa. Notably, the long-term functional impact of a microorganism that can persist in the human gut is higher to a transient one. However, to effectively study its ability to persist within the human gut, a clinical trial involving this strain will be essential to enhance our scientific understanding of PRL2012 and validate our *in vitro* findings regarding its interactions with the human host.

## Materials and methods

### Metagenome dataset selection

In this project, 4,019 publicly available datasets of human gut microbiome were retrieved from 82 cohorts of healthy individuals (Supplementary Table S1). Additionally, 9,505 samples belonging to infants (age < 3 years) were used to explore the infant-associated microbial diversity (Lugli et al., 2023).

### *B. breve* prototype selection

Complete and partial genome sequences of 166 *B. breve* strains were retrieved from the RefSeq NCBI database, representing a collection of publicly available genome sequences of this taxon. Then, the genome sequence of reference strain LMG 13208 was used to discard those strains showing an average nucleotide identity (ANI) lower than 94%, employing fastANI (Jain et al., 2018) to discard misclassified microorganisms. Furthermore, the quality of genomes was estimated for completeness and contamination using CheckM (Parks et al., 2015). High-quality genomes were then subjected to a de-replication based analysis aimed at reducing strain redundancy among bifidobacterial genome sequences using dRep v2.0 (Olm et al., 2017). Among strains displaying sequence identity >99.8%, a single reference genome was selected for further analysis, representing putative prototypes of the *B. breve* species. Then, a k-mer based analysis to explore the distribution of each putative prototype was investigated using InStrain software with a k-mer size of 23 (Olm et al., 2021). The selection of the *B. breve* prototype of the human gut was chosen using a previously validated index called AxP (Fontana et al., 2022), defined as (the average ANI value of genomes constituting the same clade) ^*^ (prevalence score of the strain in the dataset) ^*^ (100).

### Genome sequencing

The genome sequence of PRL2012 was determined by GenProbio Srl (Parma, Italy) using the MiSeq platform (Illumina, UK). Genome libraries were prepared using an Illumina Nextera XT DNA Library Preparation Kit (Illumina Inc., San Diego, CA 92122, USA). Libraries were quantified using a fluorometric Qubit quantification system (Life Technologies, USA), loaded on a 2200 TapeStation instrument (Agilent Technologies, USA), and normalized to 4 nM. Sequencing was performed using the Illumina MiSeq platform with a 600-cycles flow cell version 3 (Illumina Inc., San Diego, CA 92122, USA). PRL2012 extracted DNA was also subjected to whole-genome sequencing using the Nanopore DNA sequencing platform according to the supplier's protocol (Oxford Nanopore, UK).

### Genome assembly

Long reads were filtered by quality using the Filtlong tool (https://github.com/rrwick/Filtlong), while short reads were filtered through the fastq-mcf script (https://github.com/ExpressionAnalysis/ea-utils). Filtered fastq files of Nanopore long reads obtained from genome sequencing efforts were then used as input for genome assembly through CANU software (Koren et al., 2017). The resulting genome sequence has been polished through Polypolish (Wick and Holt, 2022) using Illumina paired-end reads. The whole process was managed by the MEGAnnotator2 pipeline (Lugli and Ventura, 2022).

### Comparative genomics

Open reading frames (ORFs) of each *B. breve* genome were predicted with Prodigal (Hyatt et al., 2010) and annotated utilizing the MEGAnnotator2 pipeline (Lugli et al., 2016; Lugli and Ventura, 2022). Proteomes were employed for a pangenome calculation using the PGAP (Zhao et al., 2012), to identify orthologs among analyzed *B. breve* strains by means of BLAST analysis (cut-off E-value, < 1^*^10^−5^; 50% identity over at least 80% of both protein sequences). The resulting output was then clustered into protein families named Clusters of Orthologous Groups (COGs) by means of MCL (graph theory-based Markov clustering algorithm), using the gene family (GF) method. Pangenome profiles were built using an optimized algorithm incorporated in the PGAP software, based on a presence/absence matrix that included all identified COGs in the 37 analyzed genomes. Furthermore, genes encoding for transporters have been annotated using the Transport Classification (TC) system (Saier et al., 2021).

### Glycobiome profiling

The proteome of PRL2012 was screened for genes predicted to encode carbohydrate-active enzymes based on sequence similarity to genes classified in the carbohydrate-active enzyme (CAZy) database (Drula et al., 2022). Thus, each gene sequence was screened for orthologs against the dbCAN2 meta server (Zhang et al., 2018) composed of 2,141,452 CDS using HMMER v3.3.2 (cut-off E-value of 1^*^10^−15^ and coverage >0.35) and DIAMOND (*E*-value < 1^*^10^−102^).

### Cultivation conditions

*B. breve* PRL2012 was cultivated in de Man-Rogosa-Sharpe (MRS) medium (Sharlau Chemie, Spain) supplemented with 0.05% (wt/vol) L-cysteine hydrochloride (Merk, Germany) and incubated at 37°C in an anaerobic cabinet (Concept 400, Ruskinn) with an anaerobic atmosphere (2.99% H_2_, 17.01% CO_2_, and 80% N_2_).

For bi-association experiments, *Bifidobacterium bifidum* PRL2010, *Bifidobacterium longum* PRL2022 and *Bifidobacterium pseudocatenulatum* LMG 10505 were cultivated as described above. In addition, *Clostridium innocuum* 107F, *Eggerthella lenta* 180F and *Ruminococcus gnavus* DSM 114966 (purchased from DSMZ-German Collection of Microorganisms and Cell Cultures GmbH) were cultivated anaerobically in yeast extract-casein hydrolysate-fatty acid (YCFA) medium in Hungate tubes at 37° C for 48 h.

### Carbohydrate growth assays

In order to extend *in silico* findings, we performed growth assays on selected carbon sources involving selected *B. breve* PRL2012 and the type strain LMG 13208. Notably, *in silico* analyses performed in this study generated predictions with regards to (carbohydrate) metabolic abilities of the above-mentioned strains and further discussed in the Results and discussion section. *B. breve* strains were cultivated overnight on a semisynthetic MRS medium supplemented with 0.05% (w/vol) L-cysteine hydrochloride at 37°C under anaerobic conditions. Subsequently, cells were diluted in MRS without glucose to obtain an OD_600nm_~1 and 15 μl of the diluted cells were inoculated in 135 μl of MRS without glucose supplemented with 1% (wt/vol) of a particular sugar in a 96-well microtiter plate and incubated in an anaerobic cabinet. Specifically, each carbohydrate was dissolved in MRS without glucose previously sterilized by autoclaving at 121°C for 15 min. Subsequently, each obtained solution was filter sterilized using a 0.2 μm filter size prior to use. Cell growth was evaluated by monitoring the optical density at 600 nm with the use of a plate reader (Biotek, VT, USA). The plate was read in discontinuous mode, with absorbance readings performed at 3 min intervals for three times after 48 h of growth, and each reading was performed following 30 s of shaking at medium speed. Cultures were grown in triplicates, and the resulting growth data were expressed as the average of these replicates. Carbohydrates tested in this study were purchased from Merck (Germany) and include arabinose, fructose, glucose, mannitol, rhamnose, ribose, sorbitol, xylose, lactose, maltose, melibiose, sucrose, trehalose, turanose, soluble starch from potato, raffinose, lactulose, cellobiose, fucose, galactose, mannose, N-acetyl-D-glucosamine, N-acetyl-D-galactosamine, inulin, amylopectin, pullulan, maltotriose, maltodextrin, glycogen, fructooligosaccharide (FOS), arabinogalactan, xylan and mucin from porcine stomach.

### *In vitro* evaluation of the interaction of PRL2012 cells with other members of the human gut microbiota

To evaluate if and how *B. breve* PRL2012 interacts with other gut microbial players, batch cultures were set up to co-cultivate the selected strain with each or a mix of six previously selected different intestinal commensals, i.e., *B. bifidum* PRL2010, *B. longum* PRL2022, *B. pseudocatenulatum* LMG 10505, *C. innocuum* 107F, *E. lenta* 180F, and *R. gnavus* DSM 114966. For bi-association experiments, overnight cultures of each microorganism were diluted in order to obtain an OD value of 1.0, as previously described (Mancabelli et al., 2021). Each culture was inoculated at 0.1% (vol/vol) into yeast extract-casein hydrolysate-fatty acid (YCFA) medium (Dostal et al., 2015; Wylensek et al., 2020; Aranda-Díaz et al., 2022). We performed six different experiments in which *B. breve* PRL2012 was inoculated with each of six different intestinal players mentioned above and one experiment where all microorganisms were cultivated together. Batch cultures were performed in triplicate and incubated under anaerobic conditions in Hungate tubes at 37°C. After 8h of incubation, cultures were centrifuged at 7000 rpm for 5 min, the supernatants were discarded, while the obtained bacterial pellets were used for RNA extraction (Scott et al., 2014; Schwab et al., 2017; Bunesova et al., 2018). Moreover, pellets were subjected to DNA extraction using the GeneElute bacterial genomic DNA kit (Sigma, Germany) following the manufacturer's instructions. Each sample was subjected to a different cycle of quantitative PCR (qPCR) using strain specific primers: Br12L_105Fw (5'-CGAAGTTCCAGTTCACCAT-3') and Br12L_105Rv (5'-GTTCTTGGCGTTCCAGATGT-3') for *B. breve* PRL2012. qPCR was performed using qPCR green master mix (PowerUp™ SYBR™ Green Master Mix for qPCR, Thermo Fisher Scientific, US) on a CFX96 system (Bio-Rad, CA, USA) following previously described protocols (Henriques et al., 2012; Milani et al., 2015a). PCR products were detected with SYBR green fluorescent dye and amplified according to the following protocol: one cycle of 50°C for 2 min, followed by one cycle of 95°C for 2, followed by 40 cycles of 95°C for 15 s and 60°C for 1 min. The melting curve was 65°C to 95°C with increments of 0.5°C/s. In each run, negative controls (no DNA) were included. A standard curve was built using the CFX96 software (Bio-Rad).

### RNA extraction

Total RNA from bacterial cells was isolated using a previously described method (Turroni et al., 2016; Milani et al., 2020). Briefly, bifidobacterial cell pellets were resuspended in 1 ml of QIAzol lysis reagent (Qiagen, Germany) in a sterile tube containing glass beads. Cells were lysed by alternating 2 min of stirring the mix on a bead beater with 2 min of static cooling on ice. These steps were repeated three times. Lysed cells were centrifuged at 12,000 rpm for 15 min, and the upper phase was recovered. Bacterial RNA was subsequently purified using the RNeasy Mini Kit (Qiagen, Germany) following the manufacturer's instructions. Then, RNA concentration and purity were evaluated using a spectrophotometer (Eppendorf, Germany).

### mRNA sequencing analysis

Total bacterial RNA (from 100 ng to 1 μg) was treated to remove rRNA by means of the QIAseq FastSelect – 5S/16S/23S following the manufacturer's instructions (Qiagen, Germany). The yield of rRNA depletion was checked using a 2200 TapeStation (Agilent Technologies, USA). Then, a whole transcriptome library for prokaryotic RNA was constructed using the TruSeq Stranded mRNA Sample preparation kit (Illumina, San Diego, USA). Samples were then loaded onto a NextSeq high output v2 kit (150 cycles) (Illumina) as indicated by the technical support guide. The obtained reads were filtered to remove low-quality reads using fastq-mcf tool (minimum mean quality 20, minimum length 100 bp) as well as any remaining ribosomal locus-encompassing reads (Milani et al., 2021). The retained reads were then aligned to the complete, closed PRL2012 genome sequence through Bowtie2 software (Langdon, 2015). Subsequently, quantification of reads mapped to individual transcripts was achieved through the htseq-counts script of HTSeq software in “union” mode (Anders et al., 2015). Raw counts were then normalized using the Trimmed Mean of M-Values (TMM) method implemented in the EdgeR package (version 3.6.1) (Robinson et al., 2010) and Log2 fold change (logFC) was used to evaluate the differences in gene expression of PRL2012 cultivated alone (reference condition), and in bi- or multi-associations (test conditions). EdgeR package was also used to identify differentially expressed genes at a false discovery rate (FDR) of 5 % and minimal logFC 1.

### Mucin adhesion assay of *B. breve* PRL2012

The effect of bifidobacterial adhesion on mucin was performed by adapting the protocol described by Valeriano et al. (2014). Briefly, 100 μL of a 1 mg mL^−1^ sterile mucin solution dissolved in a buffer saline (PBS, pH 7.4) was aliquoted into 96-well microtiters (Sarstedt, Germany) and incubated overnight at 4°C. Subsequently, each well was washed with 200 μL of PBS, rinsed, filled with 100 μL of a 20 mg mL^−1^ sterile bovine serum albumin solution, and incubated at 4°C for 2 h. Bifidobacterial growth was monitored until a concentration of 10^8^ CFU mL^−1^ was reached, after which 100 μL of the resulting bacterial suspension, previously washed and resuspended in PBS, was added in each well and incubated under anaerobic condition at 37°C for 1 h. After incubation, each well was washed two times with 200 μL of PBS to remove unbound bacteria. Then, 200 μL of 0.5% (vol/vol) Triton X-100 was added and incubated at room temperature for 2 h, under slight agitation to detach the adherent bacteria. The viable cell count expressed as CFU mL^−1^ was determined in all cases by plating on MRS medium. Each assay was performed in triplicate. Percentage adhesion was calculated as follows:


$$\begin{array}{l} \text{relative adhesion}=\left(\text{logCF}{\text{U}}_{\text{Nadhered}}/\text{logCF}{\text{U}}_{\text{Ninoculum}}\right)\times \;100 \end{array}$$

### Adhesion of *B. breve* PRL2012 to HT29-MTX cells

Bifidobacterial adhesion to HT29-MTX cells was assessed following the protocol described by Guglielmetti et al. (2008), Serafini et al. (2013). Briefly, human colorectal adenocarcinoma HT29-MTX cells (kindly provided by Professor A. Baldi, University of Milan) were cultured in Dulbecco's modified Eagle's medium (DMEM) supplemented with 10% fetal bovine serum (FBS), 2 mM glutamine, 100 μg/mL streptomycin, 100 U/mL penicillin and maintained in standard culture conditions. For the experiments, HT29-MTX cells were seeded on microscopy cover glasses previously settled into 10-cm^2^ petri dishes. Confluent cells were carefully washed twice with PBS before bacterial cells were added. *B. breve* strains, i.e., PRL2012 and LMG 13208, and *B. animalis* subsp. *lactis* Bb-12 were grown as previously described, until a concentration of 5 × 10^7^ CFU mL^−1^ was reached. The strains were then centrifuged at 3,000 rpm for 8 min, resuspended in PBS (pH 7.3), and incubated with monolayers of HT29-MTX cells. After 1 h incubation at 37°C, cultures were washed twice with 2 mL of PBS to remove unbound bacteria. The cells were then fixed with 1 mL of methanol, incubated for 8 min at room temperature, stained with 1.5 mL of Giemsa stain solution (1:20) (Sigma-Aldrich, Milan, Italy) and left in the dark for 30 min at room temperature. After two washes with 2 mL of PBS, the cover glasses were removed from the petri plate, mounted on a glass slide, and examined using a phase-contrast microscope Zeiss Axiovert 200 (objective, 100 × /1.4 oil). Adherent bacteria in 20 randomly selected microscopic fields were counted and averaged. The proportion of bacterial cells that remained attached to the HT29-MTX monolayer was determined to reflect the extent of specific host-microbe interaction. The adhesion index represents the average number of bacterial cells attached to 100 HT29-MTX cells (Guglielmetti et al., 2008; Turroni et al., 2013; Rizzo et al., 2023). A non-parametric Mann Whitney test was applied for the detection of statistically significant differences. All assays were performed at least in triplicate.

### Statistical analysis

Similarities between samples (beta-diversity) were calculated by the Bray-Curtis dissimilarity matrix based on species abundance, using the “vegdist” function on RStudio (http://www.rstudio.com/). Beta-diversity was represented through Principal Coordinate Analysis (PCoA) using the function “ape” of the R suite package (Paradis and Schliep, 2019). Moreover, the various detected bacterial species were tested and plotted on the PCoA using the “envfit” and “plot” functions from vegan through R-studios (http://www.rstudio.com/). PERMANOVA analyses were performed on RStudio using 999 permutations to estimate *p*-values for population differences in PCoA analyses with adonis2 package. Furthermore, a correlation analysis between the available metadata and the various detected bacterial species of all samples was performed through Spearman's rank correlation coefficient using “rcorr” function (https://CRAN.R-project.org/package=Hmisc), and only results that were significantly different from a statistical perspective were retained. The False Discovery Rate (FDR) correction was applied to all statistical analyses based on Benjamini and Hochberg Correction (Benjamini et al., 2001), using RStudio through “p.adjust” function.

## Data availability statement

The datasets presented in this study can be found in online repositories. The names of the repository/repositories and accession number(s) can be found in the article/Supplementary material. The updated genome sequence of *B. breve* PRL2012 was deposited in the GenBank database with the NCBI RefSeq Accession code CP006711.2.

## Ethics statement

Ethical approval was not required for the studies on humans in accordance with the local legislation and institutional requirements because only commercially available established cell lines were used.

## Author contributions

CA: Writing – original draft, Investigation, Methodology. GAL: Data curation, Investigation, Software, Writing – original draft. CT: Data curation, Software, Writing – review & editing. FF: Data curation, Software, Writing – review & editing. LM: Data curation, Software, Writing – review & editing, Validation. AV: Methodology, Writing – review & editing. RA: Methodology, Writing – review & editing. LA: Methodology, Writing – review & editing. GA: Writing – review & editing, Validation. GL: Writing – review & editing, Validation. MB: Writing – review & editing, Validation. GT: Writing – review & editing, Validation. OB: Writing – review & editing, Supervision. CM: Writing – review & editing, Supervision. DS: Writing – review & editing, Supervision. FT: Writing – review & editing, Supervision. MV: Conceptualization, Writing – review & editing, Supervision.

## References

[B1] Alessandri G. Fontana F. Tarracchini C. Rizzo S. M. Bianchi M. G. Taurino G.. (2023). Identification of a prototype human gut *Bifidobacterium longum* subsp. longum strain based on comparative and functional genomic approaches. Front. Microbiol. 14, 1130592. 10.3389/fmicb.2023.1130592 36846784 PMC9945282

[B2] Alessandri G. Ossiprandi M. C. MacSharry J. van Sinderen D. Ventura M. (2019). Bifidobacterial dialogue with its human host and consequent modulation of the immune system. Front. Immunol. 10, 2348. 10.3389/fimmu.2019.02348 31632412 PMC6779802

[B3] Anders S. Pyl P. T. Huber W. (2015). HTSeq–a Python framework to work with high-throughput sequencing data. Bioinformatics 31, 166–169. 10.1093/bioinformatics/btu638 25260700 PMC4287950

[B4] Aranda-Díaz A. Ng K. M. Thomsen T. Real-Ramírez I. Dahan D. Dittmar S.. (2022). Establishment and characterization of stable, diverse, fecal-derived in vitro microbial communities that model the intestinal microbiota. Cell Host Microbe. 30, 260–272.e5. 10.1016/j.chom.2021.12.008 35051349 PMC9082339

[B5] Benjamini Y. Drai D. Elmer G. Kafkafi N. Golani I. (2001). Controlling the false discovery rate in behavior genetics research. Behav. Brain Res. 125, 279–284. 10.1016/S0166-4328(01)00297-2 11682119

[B6] Bottacini F. O'Connell Motherway M. Kuczynski J. O'Connell K. J. Serafini F. Duranti S.. (2014). Comparative genomics of the *Bifidobacterium breve* taxon. BMC Genom. 15, 170. 10.1186/1471-2164-15-170 24581150 PMC4007704

[B7] Bottacini F. Van Sinderen D. Ventura M. (2017). Omics of bifidobacteria: Research and insights into their health-promoting activities. Biochem. J. 474, 4137–4152. 10.1042/BCJ20160756 29212851

[B8] Bozzi Cionci N. C. Baffoni L. Gaggìa F. Di Gioia D. (2018). Therapeutic microbiology: the role of bifidobacterium breve as food supplement for the prevention/treatment of paediatric diseases. Nutrients 10, 328. 10.20944/preprints201810.0328.v1 30423810 PMC6265827

[B9] Bunesova V. Lacroix C. Schwab C. (2018). Mucin cross-feeding of infant bifidobacteria and *Eubacterium hallii*. Microb. Ecol. 75, 228–238. 10.1007/s00248-017-1037-4 28721502

[B10] Cebra J. J. (1999). Influences of microbiota on intestinal immune system development. Am. J. Clin. Nutr. 69, 1046. 10.1093/ajcn/69.5.1046s 10232647

[B11] Chassard C. Lacroix C. (2013). Carbohydrates and the human gut microbiota. Curr. Opin. Clin. Nutr. Metab. Care 16, 453–460. 10.1097/MCO.0b013e3283619e63 23719143

[B12] Choi I. Y. Kim J. Kim S. H. Ban O. H. Yang J. Park M. K.. (2021). Safety evaluation of *Bifidobacterium breve* IDCC4401 isolated from infant feces for use as a commercial probiotic. J. Microbiol. Biotechnol. 31, 949–955. 10.4014/jmb.2103.03041 34024895 PMC9706084

[B13] Collado M. C. Cernada M. Baüerl C. Vento M. Pérez-Martínez G. (2012). Microbial ecology and host-microbiota interactions during early life stages. Gut Microbes 3, 21215. 10.4161/gmic.21215 22743759 PMC3463493

[B14] Dostal A. Lacroix C. Bircher L. Pham V. T. Follador R. Zimmermann M. B.. (2015). Iron modulates butyrate production by a child gut microbiota *in vitro*. mBio 6, 15. 10.1128/mBio.01453-15 26578675 PMC4659462

[B15] Drula E. Garron M. L. Dogan S. Lombard V. Henrissat B. Terrapon N.. (2022). The carbohydrate-active enzyme database: functions and literature. Nucleic Acids Res. 50, D571–D577. 10.1093/nar/gkab1045 34850161 PMC8728194

[B16] Egan M. O'Connell Motherway M. Kilcoyne M. Kane M. Joshi L. Ventura M.. (2014). Cross-feeding by *Bifidobacterium breve* UCC2003 during co-cultivation with Bifidobacterium bifidum PRL2010 in a mucin-based medium. BMC Microbiol. 14, 1–15. 10.1186/s12866-014-0282-7 25420416 PMC4252021

[B17] Fanning S. Hall L. J. van Sinderen D. (2012). Bifidobacterium breve UCC2003 surface exopolysaccharide production is a beneficial trait mediating commensal-host interaction through immune modulation and pathogen protection. Gut Microbes. 3, 420–425. 10.4161/gmic.20630 22713271

[B18] Fontana F. Alessandri G. Tarracchini C. Bianchi M. G. Rizzo S. M. Mancabelli L.. (2022). Designation of optimal reference strains representing the infant gut bifidobacterial species through a comprehensive multi-omics approach. Environ. Microbiol. 24, 5825–5839. 10.1111/1462-2920.16205 36123315 PMC10092070

[B19] Garrigues C. Johansen E. Pedersen M. B. (2010). Complete genome sequence of Bifidobacterium animalis subsp. lactis BB-12, a widely consumed probiotic strain. J. Bacteriol. 192, 2467–2468. 10.1128/JB.00109-10 20190051 PMC2863482

[B20] Guglielmetti S. Tamagnini I. Mora D. Minuzzo M. Scarafoni A. Arioli S.. (2008). Implication of an outer surface lipoprotein in adhesion of *Bifidobacterium bifidum* to Caco-2 cells. Appl. Environ. Microbiol. 74, 4695–4702. 10.1128/AEM.00124-08 18539800 PMC2519326

[B21] Henriques A. Cereija T. MacHado A. Cerca N. (2012). In silico vs in vitro analysis of primer specificity for the detection of *Gardnerella vaginalis, Atopobium vaginae* and *Lactobacillus* spp. BMC Res. Notes 5, 637. 10.1186/1756-0500-5-637 23153093 PMC3522034

[B22] Hidalgo-Cantabrana C. Delgado S. Ruiz L. Ruas-Madiedo P. Sánchez B. Margolles A.. (2017). Bifidobacteria and their health-promoting effects. Microbiol. Spectr. 5, 2016. 10.1128/microbiolspec.BAD-0010-2016 28643627 PMC11687494

[B23] Hooper L. V. (2004). Bacterial contributions to mammalian gut development. Trends Microbiol. 12, 129–134. 10.1016/j.tim.2004.01.001 15001189

[B24] Hougee S. Vriesema A. J. M. Wijering S. C. Knippels L. M. J. Folkerts G. Nijkamp F. P.. (2010). Oral treatment with probiotics reduces allergic symptoms in ovalbumin-sensitized mice: a bacterial strain comparative study. Int. Arch. Allergy Immunol. 151, 107–117. 10.1159/000236000 19752564

[B25] Hyatt D. Chen G. L. LoCascio P. F. Land M. L. Larimer F. W. Hauser L. J. (2010). Prodigal: prokaryotic gene recognition and translation initiation site identification. BMC Bioinf. 11, 119. 10.1186/1471-2105-11-119 20211023 PMC2848648

[B26] Inoue Y. Iwabuchi N. Xiao J. Z. Yaeshima T. Iwatsuki K. (2009). Suppressive effects of bifidobacterium breve strain M-16V on T-helper type 2 immune responses in a murine model. Biol. Pharm. Bull. 32, 760–763. 10.1248/bpb.32.760 19336921

[B27] Jain C. Rodriguez-R L. M Phillippy A. M. Konstantinidis K. T. Aluru S. (2018). High throughput ANI analysis of 90K prokaryotic genomes reveals clear species boundaries. Nat. Commun. 9, 641. 10.1038/s41467-018-07641-9 30504855 PMC6269478

[B28] James K. Motherway M. O. C. Bottacini F. Van Sinderen D. (2016). Bifidobacterium breve UCC2003 metabolises the human milk oligosaccharides lacto-N-tetraose and lacto-N-neo-tetraose through overlapping, yet distinct pathways. Sci. Rep 6, 560. 10.1038/srep38560 27929046 PMC5144078

[B29] Jandhyala S. M. Talukdar R. Subramanyam C. Vuyyuru H. Sasikala M. Reddy D. N.. (2015). Role of the normal gut microbiota. World J. Gastroenterol. 21, 8836–8847. 10.3748/wjg.v21.i29.8787 26269668 PMC4528021

[B30] Jungersen M. Wind A. Johansen E. Christensen J. E. Stuer-Lauridsen B. Eskesen D.. (2014). The Science behind the probiotic strain *Bifidobacterium animalis* subsp. lactis BB-12(^®^). Microorganisms 2, 92–110. 10.3390/microorganisms2020092 27682233 PMC5029483

[B31] Khodayar-Pardo P. Mira-Pascual L. Collado M. C. Martínez-Costa C. (2014). Impact of lactation stage, gestational age and mode of delivery on breast milk microbiota. J. Perinatol. 34, 599–605. 10.1038/jp.2014.47 24674981

[B32] Khoroshkin M. S. Leyn S. A. Van Sinderen D. Rodionov D. A. (2016). Transcriptional regulation of carbohydrate utilization pathways in the Bifidobacterium genus. Front. Microbiol. 7. 10.3389/fmicb.2016.00120 26903998 PMC4746261

[B33] Kiu R. Treveil A. Harnisch L. C. Caim S. Leclaire C. van Sinderen D.. (2020). *Bifidobacterium breve* UCC2003 induces a distinct global transcriptomic program in neonatal murine intestinal epithelial cells. iScience 23, 101336. 10.1016/j.isci.2020.101336 32683312 PMC7371750

[B34] Koren S. Walenz B. P. Berlin K. Miller J. R. Bergman N. H. Phillippy A. M.. (2017). Canu: scalable and accurate long-read assembly via adaptive k-mer weighting and repeat separation. Genome Res. 27, 722–736. 10.1101/gr.215087.116 28298431 PMC5411767

[B35] Langdon W. B. (2015). Performance of genetic programming optimised Bowtie2 on genome comparison and analytic testing (GCAT) benchmarks. BioData Min. 8, 1–15. 10.1186/s13040-014-0034-0 25621011 PMC4304608

[B36] Lugli G. A. Duranti S. Albert K. Mancabelli L. Napoli S. Viappiani A.. (2019). Unveiling genomic diversity among members of the species *Bifidobacterium pseudolongum*, a widely distributed gut commensal of the animal kingdom. Appl. Environ. Microbiol. 85, 1–18. 10.1128/AEM.03065-18 30737347 PMC6450028

[B37] Lugli G. A. Duranti S. Milani C. Mancabelli L. Turroni F. Alessandri G.. (2020a). Investigating bifidobacteria and human milk oligosaccharide composition of lactating mothers. FEMS Microbiol. Ecol. 96, 049. 10.1093/femsec/fiaa049 32188978

[B38] Lugli G. A. Mancabelli L. Milani C. Fontana F. Tarracchini C. Alessandri G.. (2023). Comprehensive insights from composition to functional microbe-based biodiversity of the infant human gut microbiota. NPJ Biofilms Microb. 9, 25. 10.1038/s41522-023-00392-6 37169786 PMC10175488

[B39] Lugli G. A. Milani C. Duranti S. Mancabelli L. Mangifesta M. Turroni F.. (2018). Tracking the taxonomy of the genus Bifidobacterium based on a phylogenomic approach. Appl. Environ. Microbiol. 84, 1–17. 10.1128/AEM.02249-17 29222102 PMC5795081

[B40] Lugli G. A. Milani C. Mancabelli L. Van Sinderen D. Ventura M. (2016). MEGAnnotator: a user-friendly pipeline for microbial genomes assembly and annotation. FEMS Microbiol. Lett. 363, fnw049. 10.1093/femsle/fnw049 26936607

[B41] Lugli G. A. Tarracchini C. Alessandri G. Milani C. Mancabelli L. Turroni F.. (2020b). Decoding the genomic variability among members of the *Bifidobacterium dentium* species. Microorganisms 8, 1–18. 10.3390/microorganisms8111720 33152994 PMC7693768

[B42] Lugli G. A. Ventura M. (2022). A breath of fresh air in microbiome science: shallow shotgun metagenomics for a reliable disentangling of microbial ecosystems. Microbiome Res. Rep. 1, 1–7. 10.20517/mrr.2021.07 38045646 PMC10688782

[B43] Mancabelli L. Mancino W. Lugli G. A. Argentini C. Longhi G. Milani C.. (2021). Amoxicillin-clavulanic acid resistance in the genus bifidobacterium. Appl. Environ. Microbiol. 87, 1–16. 10.1128/AEM.03137-20 33483308 PMC8091617

[B44] Mikami K. Takahashi H. Kimura M. Isozaki M. Izuchi K. Shibata R.. (2009). Influence of maternal bifidobacteria on the establishment of bifidobacteria colonizing the gut in infants. Pediatr. Res. 65, 669–674. 10.1203/PDR.0b013e31819ed7a8 19430378

[B45] Milani C. Alessandri G. Mancabelli L. Mangifesta M. Lugli G. A. Viappiani A.. (2020). Multi-omics approaches to decipher the impact of diet and host physiology on the mammalian gut microbiome. Appl. Environ. Microbiol. 86, e01864–20. 10.1128/AEM.01864-20 32948523 PMC7657629

[B46] Milani C. Andrea Lugli G. Duranti S. Turroni F. Mancabelli L. Ferrario C.. (2015a). Bifidobacteria exhibit social behavior through carbohydrate resource sharing in the gut. Sci. Rep. 5, 782. 10.1038/srep15782 26506949 PMC4623478

[B47] Milani C. Lugli G. A. Fontana F. Mancabelli L. Alessandri G. Longhi G.. (2021). METAnnotatorX2: a comprehensive tool for deep and shallow metagenomic data set analyses. mSystems 6, 21. 10.1128/mSystems.00583-21 34184911 PMC8269244

[B48] Milani C. Mancabelli L. Lugli G. A. Duranti S. Turroni F. Ferrario C.. (2015b). Exploring vertical transmission of bifidobacteria from mother to child. Appl. Environ. Microbiol. 81, 7078–7087. 10.1128/AEM.02037-15 26231653 PMC4579462

[B49] Moens F. Weckx S. De Vuyst L. (2016). Bifidobacterial inulin-type fructan degradation capacity determines cross-feeding interactions between bifidobacteria and *Faecalibacterium prausnitzii*. Int. J. Food Microbiol. 231, 76–85. 10.1016/j.ijfoodmicro.2016.05.015 27233082

[B50] Motherway M. O. C. Zomer A. Leahy S. C. Reunanen J. Bottacini F. Claesson M. J.. (2011). Functional genome analysis of *Bifidobacterium breve* UCC2003 reveals type IVb tight adherence (Tad) pili as an essential and conserved host-colonization factor. Proc. Natl. Acad. Sci. U. S. A. 108, 11217–11222. 10.1073/pnas.1105380108 21690406 PMC3131351

[B51] Neville H. J. Stevens C. Pakulak E. Bell T. A. Fanning J. Klein S.. (2013). Family-based training program improves brain function, cognition, and behavior in lower socioeconomic status preschoolers. Proc. Natl. Acad. Sci. U. S. A. 110, 12138–12143. 10.1073/pnas.1304437110 23818591 PMC3718115

[B52] Olm M. R. Brown C. T. Brooks B. Banfield J. F. (2017). dRep: a tool for fast and accurate genomic comparisons that enables improved genome recovery from metagenomes through de-replication. ISME J. 11, 2864–2868. 10.1038/ismej.2017.126 28742071 PMC5702732

[B53] Olm M. R. Crits-Christoph A. Bouma-Gregson K. Firek B. A. Morowitz M. J. Banfield J. F.. (2021). inStrain profiles population microdiversity from metagenomic data and sensitively detects shared microbial strains. Nat. Biotechnol. 39, 727–736. 10.1038/s41587-020-00797-0 33462508 PMC9223867

[B54] Paradis E. Schliep K. (2019). ape 5.0: an environment for modern phylogenetics and evolutionary analyses in R. Bioinformatics 35, 526–528. 10.1093/bioinformatics/bty633 30016406

[B55] Parks D. H. Imelfort M. Skennerton C. T. Hugenholtz P. Tyson G. W. (2015). CheckM: assessing the quality of microbial genomes recovered from isolates, single cells, and metagenomes. Genome Res. 25, 1043–1055. 10.1101/gr.186072.114 25977477 PMC4484387

[B56] Rizzo S. M. Alessandri G. Lugli G. A. Fontana F. Tarracchini C. Mancabelli L.. (2023). Exploring molecular interactions between human milk hormone insulin and bifidobacteria. Microbiol. Spectr 11, 23. 10.1128/spectrum.00665-23 37191543 PMC10269646

[B57] Robinson M. D. McCarthy D. J. Smyth G. K. (2010). edgeR: a Bioconductor package for differential expression analysis of digital gene expression data. Bioinformatics 26, 139–140. 10.1093/bioinformatics/btp616 19910308 PMC2796818

[B58] Ryan S. M. Fitzgerald G. F. Van Sinderen D. (2006). Screening for and identification of starch-, amylopectin-, and pullulan-degrading activities in bifidobacterial strains. Appl. Environ. Microbiol. 72, 5289–5296. 10.1128/AEM.00257-06 16885278 PMC1538741

[B59] Saier M. H. Reddy V. S. Moreno-Hagelsieb G. Hendargo K. J. Zhang Y. Iddamsetty V.. (2021). The transporter classification database (TCDB): 2021 update. Nucleic Acids Res. 49, D461–D467. 10.1093/nar/gkaa1004 33170213 PMC7778945

[B60] Schwab C. Ruscheweyh H. J. Bunesova V. Pham V. T. Beerenwinkel N. Lacroix C.. (2017). Trophic interactions of infant bifidobacteria and *Eubacterium hallii* during L-Fucose and fucosyllactose degradation. Front. Microbiol. 8, 95. 10.3389/fmicb.2017.00095 28194144 PMC5277004

[B61] Scott K. P. Martin J. C. Duncan S. H. Flint H. J. (2014). Prebiotic stimulation of human colonic butyrate-producing bacteria and bifidobacteria, *in vitro*. FEMS Microbiol. Ecol. 87, 30–40. 10.1111/1574-6941.12186 23909466

[B62] Sela D. A. Chapman J. Adeuya A. Kim J. H. Chen F. Whitehead T. R.. (2008). The genome sequence of Bifidobacterium longum subsp. infantis reveals adaptations for milk utilization within the infant microbiome. Proc. Natl. Acad. Sci. U. S. A. 105, 18964–18969. 10.1073/pnas.0809584105 19033196 PMC2596198

[B63] Serafini F. Strati F. Ruas-Madiedo P. Turroni F. Foroni E. Duranti S.. (2013). Evaluation of adhesion properties and antibacterial activities of the infant gut commensal Bifidobacterium bifidum PRL2010. Anaerobe 21, 9–17. 10.1016/j.anaerobe.2013.03.003 23523946

[B64] Tarracchini C. Alessandri G. Fontana F. Rizzo S. M. Lugli G. A. Bianchi M. G.. (2023). Genetic strategies for sex-biased persistence of gut microbes across human life. Nat. Commun. 14, 4220. 10.1038/s41467-023-39931-2 37452041 PMC10349097

[B65] Tojo R. Suárez A. Clemente M. G. Los Reyes-Gavilán D. Margolles C. G. Gueimonde A.. (2014). Intestinal microbiota in health and disease: role of bifidobacteria in gut homeostasis. World J. Gastroenterol. 20, 15163–15176. 10.3748/wjg.v20.i41.15163 25386066 PMC4223251

[B66] Turroni F. Bottacini F. Foroni E. Mulder I. Kim J. H. Zomer A.. (2010). Genome analysis of *Bifidobacterium bifidum* PRL2010 reveals metabolic pathways for host-derived glycan foraging. Proc. Natl. Acad. Sci. U. S. A. 107, 19514–19519. 10.1073/pnas.1011100107 20974960 PMC2984195

[B67] Turroni F. Foroni E. Serafini F. Viappiani A. Montanini B. Bottacini F.. (2011a). Ability of Bifidobacterium breve to grow on different types of milk: exploring the metabolism of milk through genome analysis. Appl. Environ. Microbiol. 77, 7408–7417. 10.1128/AEM.05336-11 21856831 PMC3194849

[B68] Turroni F. Milani C. Duranti S. Ferrario C. Lugli G. A. Mancabelli L.. (2018). Bifidobacteria and the infant gut: an example of co-evolution and natural selection. Cell. Mol. Life Sci. 75, 103–118. 10.1007/s00018-017-2672-0 28983638 PMC11105234

[B69] Turroni F. Milani C. Duranti S. Mancabelli L. Mangifesta M. Viappiani A.. (2016). Deciphering bifidobacterial-mediated metabolic interactions and their impact on gut microbiota by a multi-omics approach. ISME J. 10, 1656–1668. 10.1038/ismej.2015.236 26859770 PMC4918443

[B70] Turroni F. Peano C. Pass D. A. Foroni E. Severgnini M. Claesson M. J.. (2012). Diversity of bifidobacteria within the infant gut microbiota. PLoS ONE 7, 36957. 10.1371/journal.pone.0036957 22606315 PMC3350489

[B71] Turroni F. Serafini F. Foroni E. Duranti S. Motherway M. O. C. Taverniti V.. (2013). Role of sortase-dependent pili of *Bifidobacterium bifidum* PRL2010 in modulating bacterium-host interactions. Proc. Natl. Acad. Sci. U. S. A. 110, 11151–11156. 10.1073/pnas.1303897110 23776216 PMC3703987

[B72] Turroni F. van Sinderen D. Ventura M. (2011b). Genomics and ecological overview of the genus Bifidobacterium. Int. J. Food Microbiol. 149, 37–44. 10.1016/j.ijfoodmicro.2010.12.010 21276626

[B73] Turroni F. van Sinderen D. Ventura M. (2021). Bifidobacteria: insights into the biology of a key microbial group of early life gut microbiota. Microbiome Res. Rep. 1, 2. 10.20517/mrr.2021.02 38045555 PMC10688781

[B74] Valeriano V. D. Parungao-Balolong M. M. Kang D. K. (2014). *In vitro* evaluation of the mucin-adhesion ability and probiotic potential of Lactobacillus mucosae LM1. J. Appl. Microbiol. 117, 485–497. 10.1111/jam.12539 24807045

[B75] Ventura M. O'Flaherty S. Claesson M. J. Turroni F. Klaenhammer T. R. van Sinderen D.. (2009). Genome-scale analyses of health-promoting bacteria: probiogenomics. Nat. Rev. Microbiol. 7, 61–71. 10.1038/nrmicro2047 19029955

[B76] Ventura M. Turroni F. van Sinderen D. (2012). Probiogenomics as a tool to obtain genetic insights into adaptation of probiotic bacteria to the human gut. Bioeng. Bugs 3, 73–79. 10.4161/bbug.18540 22095053 PMC3357336

[B77] Wick R. R. Holt K. E. (2022). Polypolish: short-read polishing of long-read bacterial genome assemblies. PLoS Comput. Biol. 18, 9802. 10.1371/journal.pcbi.1009802 35073327 PMC8812927

[B78] Wylensek D. Hitch T. C. A. Riedel T. Afrizal A. Kumar N. Wortmann E.. (2020). A collection of bacterial isolates from the pig intestine reveals functional and taxonomic diversity. Nat. Commun. 11, 6389. 10.1038/s41467-020-19929-w 33319778 PMC7738495

[B79] Zhang H. Yohe T. Huang L. Entwistle S. Wu P. Yang Z.. (2018). dbCAN2: a meta server for automated carbohydrate-active enzyme annotation. Nucleic Acids Res. 46, W95–W101. 10.1093/nar/gky418 29771380 PMC6031026

[B80] Zhao Y. Wu J. Yang J. Sun S. Xiao J. Yu J.. (2012). PGAP: pan-genomes analysis pipeline. Bioinformatics 28, 416–418. 10.1093/bioinformatics/btr655 22130594 PMC3268234

