# Supplementary material for: Genomic and ecological approaches to identify the Bifidobacterium breve prototype of the healthy human gut microbiota
Source: Front Microbiol. 2024 Feb 15;15:1349391. doi: 10.3389/fmicb.2024.1349391 (PMC10902438; doi:10.3389/fmicb.2024.1349391)
Supplement: Supplementary file 2 [file Data_Sheet_1.docx]

**Genomic and ecological approaches to identify the *Bifidobacterium breve* prototype of the healthy human gut microbiota**

**Results and discussion**

***In vitro* evaluation of tolerance of *B. breve* PRL2012 strain toward human gut challenges.** One of the significant physiological characteristics needed for bifidobacteria to thrive in their natural environment includes their ability to withstand and overcome the unfavourable and challenging conditions they face during their passage toward the large intestine. (Bottacini et al., 2014; Alessandri et al., 2021). Thus, we performed *in vitro* experiments that mimic the exposure to the osmotic stress, biliary salts, and pH reduction during their passage through the stomach (Serafini et al., 2013). In this context, *Bifidobacterium breve* PRL2012 was exposed to different concentrations of NaCl (2%, 6% and 10%) and Oxgall (0.5%, 1% and 2%) for 3h (Yasmin et al., 2020), after which cell viability was monitored through flow cytometry. Interestingly, PRL2012 showed a survival rate > 59% for the 2% NaCl, which is in line with that observed for other probiotic strains (Serafini et al., 2013). Conversely, PRL2012 displayed a reduced viability of ⁓15%, suggesting a low tolerance toward intense osmotic stress (Table S5). Nevertheless, *B. breve* PRL2012 showed a survival rate of 42.6% at the lowest concentration of Oxgall, *i.e.*, 0.5%. Overall, these assays suggest that *B. breve* PRL2012 could resist biliary salts since the physiological bile salt concentration in the human gut is estimated to be from 0.3% to 0.4% (Russell et al., 2011). Another hostile environment condition that bifidobacteria may face is the acidic environment. In particular, PRL2012 displayed a reduced survival rate, with a percentage value ranging from 14% to 16% at different pH values tested after 2h of cultivation (Table S5). Despite the reduced viability of PRL2012, a survival rate of ⁓10% may indicate that from the initial inoculum of 10^8^ cells/mL, about 10^7^ cells/mL can resist acidic conditions, as previously reported (Alessandri et al., 2023). Accordingly, previous observations highlighted reduced survival rates in the acid challenge assays conducted at pH 2.0 and pH 3.0 (Serafini et al., 2013; Achi and Halami, 2019; Yasmin et al., 2020). Overall, *in vitro* survival experiments conducted with PRL2012 to simulate the harsh conditions of the gastrointestinal tract have emphasized the strain resilience towards stressful conditions typically found in the intestinal environment.

**Materials and Methods**

**pH, sodium chloride, and bile salts tolerance tests.** To evaluate the ability of the selected strains to tolerate various pH levels, *B. breve* PRL2012 was cultivated in 10 mL of MRS broth at 37°C under anaerobic conditions to reach a final concentration of 10^8^ cells/ml. Subsequently, cells were centrifuged at 3,000 rpm for 8 min, washed with phosphate buffered saline (PBS, pH 6.5) and resuspended in 10 mL of MRS broth whose pH was adjusted to pH 2.0, pH 3.0, or pH 4.0 with the addition of HCl. Cells were incubated under anaerobic conditions at 37°C for 2h, as previously described (Yasmin et al., 2020). The same procedure was performed to assess the ability of bifidobacteria to tolerate different NaCl (2%, 6%, and 10%) or bile salts (Sigma Aldrich, US) (0.5%, 1%, and 2%) concentrations with an exposure of 3h to these stressful conditions, as previously reported (Yasmin et al., 2020). All experiments were carried out in triplicate and a control sample was obtained by inoculating bifidobacterial cells in MRS broth. After incubation, cell viability was evaluated by means of the LIVE/DEAD BacLight Bacterial Viability kit (ThermoFisher Scientific, USA) and an Attune NxT flow cytometer (ThermoFisher Scientific,USA).

**Flow cytometry bacterial viability assay.** Following exposure to acidic environment, or various bile salts or NaCl concentrations, a 10-fold serial dilution in Phosphate Buffered Solutions (PBS) was obtained from each tested condition. The diluted cells were then used for a flow cytometry cell viability assay using the fluorescent dyes SYTO9 (3.34 mM) and PI (20 mM) of the LIVE/DEAD BacLight Bacterial Viability kit (ThermoFisher Scientific, USA), following the manufacturer’s protocol (Manual of the LIVE/DEAD BacLight Bacterial Viability and counting kit, ThermoFisher Scientific, USA). Briefly, two aliquots of 1 mL of bacterial cell dilution (1:1000) were harvested by centrifugation at 3,000 rpm for 8 min and washed with PBS. Subsequently, one of the two aliquots of bacterial suspension was exposed to 70% isopropyl alcohol and kept on ice for 1h to permeabilize cell membranes and induce cell death, while the other 1 mL aliquot was maintained in PBS to preserve cell viability. Subsequently, 1.5 µl of a specific dye was added to samples for single staining assay, while for the double staining assay 1.5 µl of both dyes were added to samples. Once stained, samples were incubated in the dark for 15 min at room temperature. Furthermore, while single-stained controls were used for instrument parameter adjustment, non-stained cells were used as a background control. Cell viability assay was performed with the Attune NxT flow cytometer (ThermoFisher Scientific, USA), and all data were analyzed with the Attune NxT flow cytometer software.

**References**

Achi, S. C., and Halami, P. M. (2019). In Vitro Comparative Analysis of Probiotic and Functional Attributes of Indigenous Isolates of Bifidobacteria. *Curr Microbiol* 76, 304–311. doi: 10.1007/s00284-018-1615-9.

Alessandri, G., Fontana, F., Tarracchini, C., Rizzo, S. M., Bianchi, M. G., Taurino, G., et al. (2023). Identification of a prototype human gut Bifidobacterium longum subsp. longum strain based on comparative and functional genomic approaches. *Front Microbiol* 14. doi: 10.3389/FMICB.2023.1130592.

Alessandri, G., van Sinderen, D., and Ventura, M. (2021). The genus bifidobacterium: From genomics to functionality of an important component of the mammalian gut microbiota running title: Bifidobacterial adaptation to and interaction with the host. *Comput Struct Biotechnol J* 19, 1472–1487. doi: 10.1016/J.CSBJ.2021.03.006.

Bottacini, F., Ventura, M., Sinderen, D. van, and Motherway, M. O. C. (2014). Diversity, ecology and intestinal function of bifidobacteria. *Microb Cell Fact* 13. doi: 10.1186/1475-2859-13-S1-S4.

Russell, D. A., Ross, R. P., Fitzgerald, G. F., and Stanton, C. (2011). Metabolic activities and probiotic potential of bifidobacteria. *Int J Food Microbiol* 149, 88–105. doi: 10.1016/J.IJFOODMICRO.2011.06.003.

Serafini, F., Strati, F., Ruas-Madiedo, P., Turroni, F., Foroni, E., Duranti, S., et al. (2013). Evaluation of adhesion properties and antibacterial activities of the infant gut commensal Bifidobacterium bifidum PRL2010. *Anaerobe* 21, 9–17. doi: 10.1016/J.ANAEROBE.2013.03.003.

Yasmin, I., Saeed, M., Khan, W. A., Khaliq, A., Chughtai, M. F. J., Iqbal, R., et al. (2020). In vitro Probiotic Potential and Safety Evaluation (Hemolytic, Cytotoxic Activity) of Bifidobacterium Strains Isolated from Raw Camel Milk. *Microorganisms* 8. doi: 10.3390/MICROORGANISMS8030354.
