# Supplementary material for: Genomic and ecological approaches to identify the Bifidobacterium breve prototype of the healthy human gut microbiota
Source: Front Microbiol. 2024 Feb 15;15:1349391. doi: 10.3389/fmicb.2024.1349391 (PMC10902438; doi:10.3389/fmicb.2024.1349391)
Supplement: Supplementary file 3 [file Image_1.PDF]

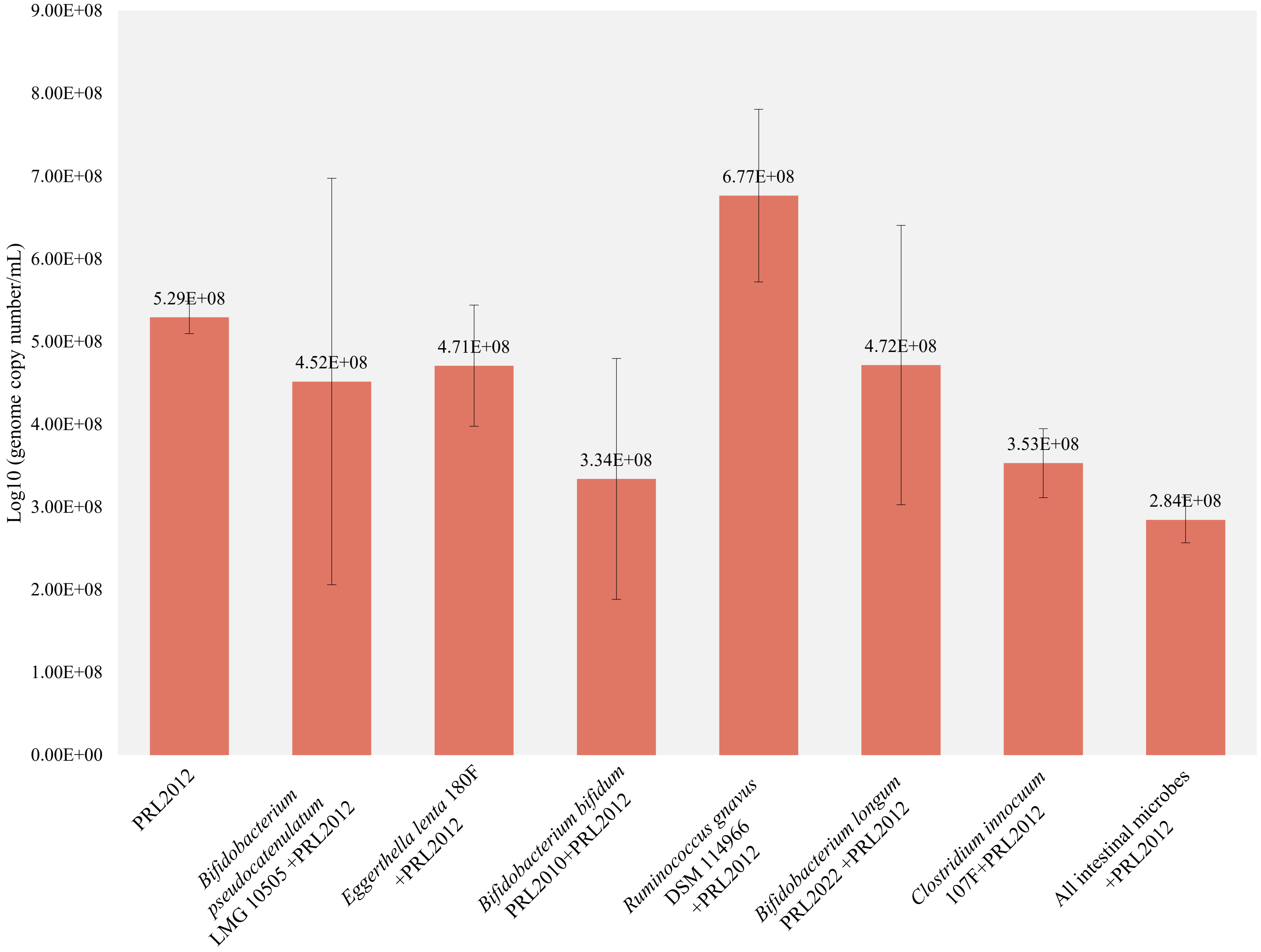

Figure S1. Quantitative PCR evaluation of the numerical load of *B. breve* PRL2012 in bi- and multi-association assays. The graph reports the average abundances of the prototype in the different co-cultivation assays.
